# Parents of children with disabilities: A systematic review of parenting interventions and self-efficacy

**DOI:** 10.4102/ajod.v7i0.437

**Published:** 2018-10-17

**Authors:** Ameer S.J. Hohlfeld, Michal Harty, Mark E. Engel

**Affiliations:** 1 Cochrane South Africa, South African Medical Research Council, South Africa; 2 Department of Health and Rehabilitation Sciences, University of Cape Town, South Africa; 3 Department of Medicine, University of Cape Town, South Africa

## Abstract

**Background:**

An increasing body of empirical evidence suggests that early intervention has positive outcomes for parents of children with neurodevelopmental disabilities. Parental self-efficacy has been used as an outcome measure in some empirical studies; however, there is a lack of evidence of the impact of parent training programmes on parenting self-efficacy beliefs.

**Objectives:**

This systematic review sought to assess the effectiveness of parenting interventions to increase parental self-efficacy levels in parents of young children with neurodevelopmental disabilities.

**Method:**

We conducted a broad literature search, which included grey literature, such as dissertations and unpublished conference presentations, to identify all relevant prospective studies reporting on our study objective. Articles were selected for inclusion using predefined criteria and data were extracted onto a purposely designed data extraction form. Twenty-five articles met our search criteria. We extracted parenting self-efficacy scores before, and on, completion of parenting interventions and performed a meta-analysis using standardised mean difference. We also conducted a risk of bias assessment for all the included studies.

**Results:**

Parent training programmes resulted in a statistically significant increase in parental self-efficacy levels (standardised mean difference, 0.60 [95% confidence interval {CI}, 0.38–0.83]; I2, 74%) relative to baseline measurements. Parents of children younger than 5 years demonstrated the highest increase in levels of parental self-efficacy after parenting interventions. Furthermore, this review showed that psychologists and other healthcare practitioners are successfully able to implement training programmes that enhance parenting self-efficacy.

**Conclusion:**

Parent training programmes are effective in increasing parental self-efficacy in parents of children with neurodevelopmental disabilities.

## Introduction

An increasing body of empirical evidence suggests that early intervention has positive outcomes for parents of children with neurodevelopmental disabilities (Guralnick 2017). Early intervention leads to an increase in developmental, social and functional outcomes for children (Dunst 2007; Guralnick 2017). Furthermore, there are numerous psychosocial benefits for parents, including an increase in parental empowerment, a decrease in parental stress and the improvement of parental self-efficacy (PSE) levels (Barlow, Coren & Stewart-Brown 2002). Consequently, lack of access to early intervention has been proposed as one explanation for why low- and middle-income (LAMI) countries have fallen short of effectively addressing Millennium Development Goals relating to child health (Samuels, Slemming & Balton 2012). In addition, many LAMI countries lack sufficiently skilled health practitioners to initiate and sustain such early interventions (Einfeld et al. 2012; Samuels et al. 2012).

Parents have an important role to play in a child’s psychosocial development (Kagan 1999). Consequently, a number of parenting interventions for families of young children with neurodevelopmental disabilities have been designed and evaluated globally over the past few decades (Kaminski et al. 2008; Salas & Cannon-Bowers 2001). These interventions are designed to improve a parent’s ability to successfully parent their children, through training, support or education, and the main goal is to influence the parent’s psychosocial well-being (Mejia, Calam & Sanders 2012). The majority of these programmes consist of skills training, parent education, parent support and/or parent coaching, and as a result they are said to be focused on the provision of knowledge (parent support) or techniques (parent-mediated intervention) (Bearss et al. 2015). The primary aims of these interventions are to reduce the impact of the challenges faced by the family of children with disabilities through teaching parents new knowledge and skills to reduce the child’s behavioural, emotional and developmental difficulties (Reichow et al. 2013). The methods of delivery of such training may include large seminar delivery, small group programmes and individual coaching sessions. The formats include telephone-assisted programmes, face-to-face programmes, self-directed programmes and online parenting programmes. The effectiveness of these programmes is not solely reliant on the delivery methods utilised, or content taught, but rather on the types of activities that are incorporated into the programmes (Kaminski et al. 2008; Woods et al. 2011). According to Kaminski et al. (2008), intervention teaching methods that included practising new skills with their own child and role play demonstrated the greatest effect size. Through these types of teaching activities, parents are taught intervention techniques that can be incorporated into their daily routines. This makes the impact of the intervention more sustainable compared to clinician-implemented interventions (Sanders & Kirby 2012; Strauss et al. 2013).

Researchers with a focus on the psychosocial development of children with developmental disorders indicate that PSE may have an important role to play in the development of a child (Coleman & Karraker 2003; Jones & Prinz 2005; Kendall & Bloomfield 2005; Montigny & Lacharité 2005). The PSE construct is primarily grounded in Bandura’s social-cognitive theory and has been defined as the belief in one’s own abilities to arrange and carry out tasks or actions to yield a specific achievement (Bandura 1977; 1989; 1997; Bandura & Walters 1977). A high level of PSE will cause parents to think and act in ways that will optimise the developmental outcomes of their children (Reichow et al. 2013). In other words, parents who face numerous stressors, but have high levels of PSE, are still able to facilitate positive developmental experiences for their children (Elder 1995). Consequently, developers of parenting interventions have paid considerable attention to the mechanisms whereby PSE beliefs can be enhanced (Bloomfield & Kendall 2007; Hudson et al. 2003; Jones & Prinz 2005; Sanders & Woolley 2005).

**FIGURE 1:**
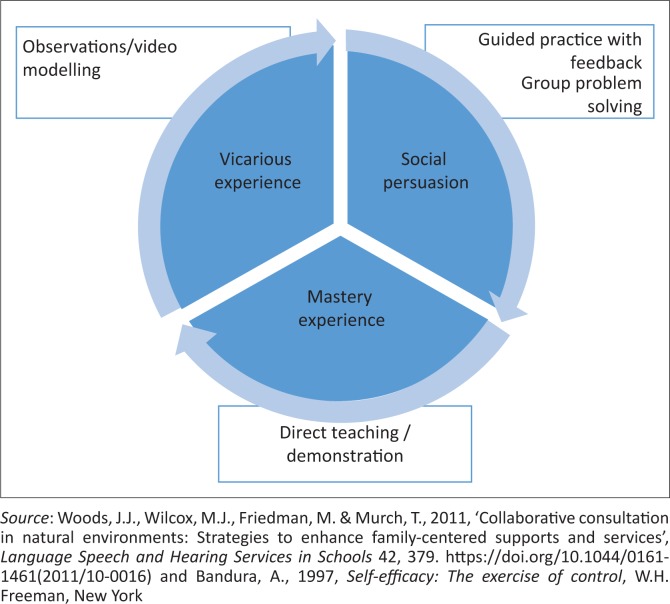
Parental self-efficacy sources frequently used in parent training programmes.

There are four primary methods in which self-efficacy can be modified (Bandura 1989). These methods serve to either enhance or decrease perceived levels of PSE. The first and most important method is that of enactive mastery (personal) experience. This results from prior accomplishment in certain activities. Enhancing PSE levels is thus achieved by allowing parents to experience success in situations that they previously found challenging (Bandura 1977). A second, likely method for improving personal self-efficacy is through the use of vicarious experiences. The individuals learn by observing challenging activities carried out by competent models, allowing them to re-evaluate their own mastery capabilities in relation to similar challenges they would encounter. It is especially useful when individuals see themselves as being similar to the observed model (Bandura 1997). Thus, having group discussions with other parents facing similar challenges, or watching videos or live parent models carrying out challenging tasks, are activities that may enhance PSE levels. A third mechanism to improve self-efficacy beliefs is the use of verbal and social persuasion, whereby others provide informed verbal feedback of an individual’s capabilities pertaining to a certain task (Bandura 1997; Woods et al. 2011). Encouragement from others is believed to be useful in improving self-efficacy and skill, whereas discouragement has the opposite effect (Bandura 1986). Within parenting programmes, feedback or coaching from the interventionists may provide this source of modification. The fourth way self-efficacy beliefs can be modified is through emotional and physiological arousal. Parents may experience stressful physiological responses that include increased stress, anxiety and/or fatigue, which make it harder to experience success (Bandura 1986). Therefore, reducing negative emotional arousal to subjective fears (through increased knowledge or skills, or access to necessary formal and informal support) would subsequently enhance performance and improve perceived self-efficacy (Bandura 1986). Figure 1 is a visual representation of common intervention activities and how they may influence self-efficacy beliefs. However, as programmes typically aim to decrease stress as an outcome for their intervention, based on the cumulative benefits of the other activities rather than the inclusion of a specific intervention activity (like mindfulness), this modifier is not included in Figure 1.

To our knowledge, there have been no systematic reviews of randomised controlled trials (RCTs) to assess the effects of parent training interventions on PSE for parents with young children that have neurodevelopmental disabilities. Through a systematic review of existing studies the primary objective was to assess the immediate change in PSE levels following parent training programmes for parents in the intervention arms of the included studies. The secondary objectives were to compare the change in PSE levels:

- for interventions directed at parents of children younger than the age of 5 years and studies directed at parents of children 5 years and older,
- for trademarked or copyrighted interventions and those without licencing,
- for studies administered by a psychologist and those that were implemented by other healthcare practitioners,
- and to conduct a moderator analysis (assess heterogeneity) and risk of bias assessment to compare the treatment effects across the different kinds of parent training programmes.

### Hypotheses

We hypothesised that there would be a significant positive effect size for PSE levels when combining all included studies. Furthermore, we predicted a larger effect size associated with licenced interventions than non-licenced interventions, as well as greater gains in PSE levels in studies targeting parents of children younger than 5 years of age compared to those targeting parents of children older than 5 years. Typical developmental milestones are well documented for children from 0 to 5 years. Consequently, skills-based parent training for parents of children with neurodevelopmental disabilities typically focus on teaching parents to facilitate their child’s development, using these milestones as guidelines. However, from age 5, many children in developed country contexts will be following a more academic curriculum in their educational context rather than a developmental curriculum. Parent training for this group of parents often targets a wider range of topics. Consequently, we hypothesised that the more focused programmes targeting parents of young children under five would have a greater impact on PSE than the programmes for parents of school-aged children, which are more heterogeneous in content. Given the multidisciplinary nature of early intervention services in developed country contexts and the nature of the activities that enhance PSE (see Figure 1), we hypothesised that any member of a multidisciplinary team should be able to implement a parent intervention that would enhance PSE.

## Methods

### Eligibility criteria

Studies selected for this review needed to meet the following inclusion criteria:

- The study needed to be an RCT using parent training interventions for parents with children diagnosed with neurodevelopmental disabilities.
- Caregivers needed to be biological parents of children (aged between 0 and 10 years) with established neurodevelopmental disabilities, including, but not limited to, an autism spectrum disorder (ASD), cerebral palsy, Down syndrome, multiple and/or significant disabilities and attention deficit hyperactivity disorder (ADHD), which is now included in the DSM-5 as a neurodevelopmental disability). The parenting skills needed to parent a young child will differ from those needed to parent a preadolescent. Preadolescence is generally defined as the period between 10 and 13 years of age. Consequently, we set the upper limit for child’s age to 10 years.
- Interventions needed to address elements of a child’s psychosocial development through parent support, training, education and/or coaching.
- The control groups needed to receive either no intervention or care as usual.
- Programmes needed to report on parental outcomes that fell under the PSE construct (we included the terms ‘parental competence’ and ‘parental confidence’ under this construct).
- The study needed to state the means, standard deviations and sample sizes in the publication or in response to a request made to the corresponding author of the publication.

Studies were excluded if:

- PSE levels were not measured,
- wrong study design,
- children were too old,
- wrong or no neurodevelopmental disability,
- intervention not described,
- full-text articles were not accessible to the researchers and/or corresponding authors were unable to provide data in time.

### Search strategy

**FIGURE 2:**
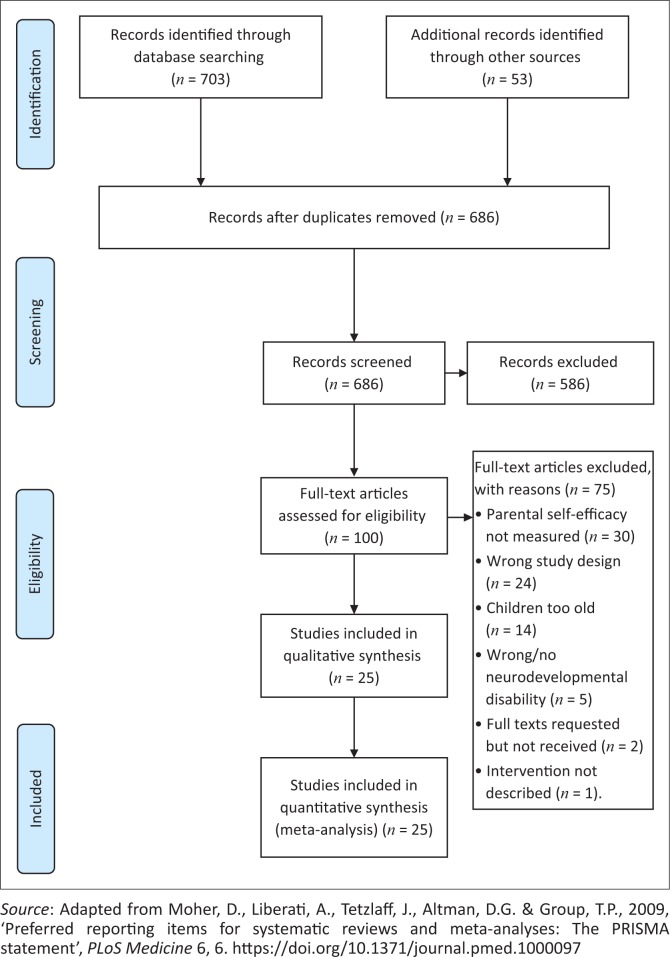
Preferred Reporting Items for Systematic Review and Meta-Analyses (PRISMA) flow chart presenting the documentation and selection of included studies in the systematic review.

Relevant studies were obtained using various strategies; an example of the search strategy used can be found in the Appendix. Two authors, Ameer Hohlfeld (A.H.) and Michal Harty (M.H.), extensively searched databases, without any language or time limitations. An updated search was conducted in August 2017. The databases searched were EMBASE, PsycINFO, PubMed, Academic Search Premier, Africa-wide Information, Cumulative Index to Nursing and Allied Health, Education Resources Information Center, Health Source (consumer edition), PsycARTICLES, Google Scholar, Dissertation Abstracts International, and the Cochrane Library (Cochrane Database of Systematic Reviews, Cochrane Central Register of Controlled Trials and Cochrane Methodology Register). Using unlimited truncation characters for each database, we used the following search strategy after determining key medical subject heading terms for each of the inclusion criteria. We supplemented the above searches with a manual search of Google Scholar and other grey literature sites. In addition, we searched reference lists of included studies to identify any missing articles, abstracts and conference proceedings, which we then requested from the authors. A.H. then revised all relevant material obtained from the search. After reading the titles and abstracts of the identified studies, we retrieved the full-text studies for every citation potentially meeting inclusion criteria. Both A.H. and M.H. revised the full-text articles using a predesigned study eligibility form to decide on the inclusion status (Figure 2).

### Data extraction

A.H. and M.H. independently extracted the data using a homogenous data extraction form, which they then cross-checked. M.E. settled discrepancies through discussion where necessary. Information extracted from the studies included country in which the study was conducted, study design, sample size, child diagnosis, mean age of the child in years and standard deviation, target parent participating in the intervention, name of the parenting intervention programme, coach or trainer administering the intervention and the tool used to measure PSE. We extracted means, standard deviations and sample sizes for each relevant intervention group measuring PSE for the analysis. Only the baseline scores and first recorded post-intervention PSE scores were extracted. Where possible we only extracted PSE scores from studies using standardised interventions if the study also tested modified or enhanced versions of the interventions.

### Data analysis

The standardised mean difference (SMD) was used to assess the overall change in PSE levels because studies used different scales to measure the mean change in PSE levels (Higgins 2009). We calculated the *I*^2^ statistic for each analysis as a measure of the proportion of the overall variation that is attributable to between-study heterogeneity (Hozo, Djulbegovic & Hozo 2005). Data were analysed using Review Manager 5.3 (The Cochrane Collaboration 2014). The outcomes (PSE, parenting competence, parenting confidence) were considered as continuous variables. In addition, meta-analyses were performed on each of the subgroups. Where significant heterogeneity was found, the random-effects model was used.

For the PSE measures, some studies combined the subscales scores producing a Parenting Sense of Competence (PSOC) total score (*n* = 7), while others reported the scores on the PSOC efficacy subscale separately (*n* = 9). For the self-efficacy tools (such as the PSOC and the Parenting Tasks Checklist, PTC) that summed separate subscale scores into a total score, only the efficacy subscale scores were extracted. Where these subscale scores were not provided, we used the total score for the scale. Where studies evaluated more than one format of the intervention, we extracted data from the standard interventions and not the adapted formats.

### Risk of bias

The Preferred Reporting Items for Systematic Review and Meta-Analyses (PRISMA) statement suggests that methods describing the assessment for risk of bias be included in meta-analyses or systematic reviews (Moher et al. 2009). We individually inspected specific components in each included study for risk of bias: selection of participants for each study, sequence generation and randomisation, allocation concealment, blinding, incomplete outcome data or missing data (attrition bias), selective outcome reporting and other sources of bias. Each component of the risk of bias assessment was scored as having a high, low or unclear risk of bias according to established methods (Higgins & Altman 2008). In the event of a disagreement between A.H. and M.H., consensus was determined through consultation and discussion with M.E.

## Ethical considerations

Ethics approval is not required for this study, given that systematic reviews draw on secondary publicly available data from published studies.

## Results

### Description of studies

We obtained 1624 titles and abstracts from electronic databases and trial registries. An additional 53 references were found through manually searching the reference lists of included studies. For two of these the full-text version could not be accessed and the authors were thus contacted. Therefore, a total of 1677 studies were retrieved and, once duplicate studies were removed, 456 studies remained. A further 356 articles were excluded based on examination of title and abstracts, after which 100 articles were potentially eligible for inclusion, pending full-text assessment. A native French speaker translated a French language article. Finally, 25 articles met our inclusion criteria, of which 3 studies were not published. Figure 2 depicts a flow diagram of the literature search results.

Table 1 summarises the characteristics of the included studies. There were 1697 families who participated in the studies; the sample sizes ranged from 11 to 305. Of the 25 studies, the majority of child diagnoses included ADHD and/or conduct disorder or non-compliant behaviour problems (13 studies) and ASD (8 studies). The remainder consisted of non-specific developmental disorders (3 studies) and cerebral palsy (1 study). It is interesting to note the lack of RCTs measuring PSE for conditions such as cerebral palsy, which is fairly prevalent, particularly in LAMI countries.

**TABLE 1:** Characteristics of randomised controlled trials conducted globally meeting inclusion criteria.

Study authors (year)	Country	Sample size	Programme type	Target condition of children	Child mean age (range)	Target parent	Coach/trainer	Outcome measure
Au et al. (2014)	Hong Kong	17	Triple-P (SSTP, group)	ADHD, CD	7.69 (5–10)	Non-specific	Psychologist	PSOC (total)
Azevedo et al. (2013)	Portugal	100	IY	ADHD	4.65 (3–6)	Mothers	Psychologist	PSOC (total and efficacy)
Bor, Sanders and Markie-Dadds (2002)	Australia	87	Triple-P (standard)	ADHD, CD	3.42 (3–4)	Mothers	Psychologist	PSOC (total)
Dittman et al. (2016)	New Zealand and Australia	85	Triple-P (discussion group)	NBP	3.63 (3–5)	Non-specific	Psychologist	PTC setting and PTC behavioural
Cassidy (2001)	Australia	17	Triple-P (SSTP, self-directed)	NSDD	4.38 (2–7)	Mothers	Non-psychologist	PSOC (total)
Connell, Sanders and Markie-Dadds (1997)	Australia	23	Triple-P (self-help)	ADHD, CD	4.27 (2–6)	Mothers	Non-psychologist	PSOC (total and efficacy)
Estes et al. (2014)	USA	82	P-ESDM	ASD	1.75 (1–2)	Non-specific	Psychologist	PSOC (total and efficacy)
Frank, Keown and Sanders (2015)	New Zealand	42	Triple-P (group)	CD	5.55 (3–8)	Non-specific	Non-psychologist	PTC setting & PTC behavioural
Franke, Keown and Sanders (2016)	New Zealand	53	Triple-P (online)	ADHD	4.0 (3–4)	Non-specific	Psychologist	PSOC (efficacy)
Gardner, Burton and Klimes (2006)	UK	76	IY	ADHD, CD	5.9 (2–9)	Non-specific	Non-psychologist	PSOC (total)
Grahame et al. (2015)	UK	45	MRB	ASD	5.13 (3–7)	Non-specific	Psychologist	Parent Self-Efficacy
Harrison (2006)	Australia	28	Triple-P (SSTP group)	NSDD	3.5 (1.5–5)	Non-specific	Non-psychologist	PSOC (total and efficacy)
Ingersoll et al. (2016)	USA	28	ImPACT Online	ASD	3.65 (1.6–6.08)	Non-specific	Non-psychologist	PSOC (total)
Leung et al. (2003)	Hong Kong	69	Triple-P (group)	ADHD, CD	4.23 (3–7)	Non-specific	Non-psychologist	PSOC (total and efficacy)
Markie-Dadds and Sanders (2006)	Australia	41	Triple-P (self-help)	ADHD, CD	3.91 (2–6)	Non-specific	Psychologist	PSOC (efficacy)
Plant and Sanders (2007)	Australia	74	Triple-P, SSTP (standard)	NSDD	4.59 (<6)	Non-specific	Psychologist	PSOC (total)
Poslawsky et al. (2015)	Netherlands	78	VIPP-AUTI	ASD	3.58 (1.33–5.08)	Non-specific	Non-psychologist	PEQ
Reitzel et al. (2013)	Canada	15	FBST†	ASD	5.03 (3.17–6.83)	Non-specific	Non-psychologist	PSOC (total)
Roberts et al. (2011)	Australia	85	Building Blocks©	ASD	3.52 (2.2–5)	Mothers	Non-psychologist	PPQ (confidence)
Sanders et al. (2000)	Australia	305	Triple-P (standard)	ADHD, CD	3.41 (3–4)	Mothers	Psychologist	PSOC (total)
Sanders, Baker and Turner (2012)	Australia	116	Triple-P (online)	ADHD, CD	4.67 (2–9)	Non-specific	Non-psychologist	PTC setting and PTC behavioural
Sonuga-Barke et al. (2001)	UK	78	PT	ADHD, CD	± 3 years (range not reported)	Mothers	Non-psychologist	PSOC (efficacy)
Susman (2012)	Israel	30	Education intervention package†	Cerebral Palsy	3.67 (1.5–6)	Non-specific	Non-psychologist	Caregiving self-efficacy
Tellegen and Sanders (2014)	Australia	64	Triple-P (PCSSTP)	ASD	5.68 (2–9)	Non-specific	Psychologist	PTC setting and PTC behavioural
Whittingham et al. (2009)	Australia	59	Triple-P (SSTP)	ASD	5.91 (2–9)	Non-specific	Psychologist	PSOC (efficacy)

† , Non-licenced intervention.

SSTP, Stepping Stones Triple-P; ADHD, attention deficit/hyperactive disorder; CD, conduct disorder; PSOC, Parenting Sense of Competence; IY, the Incredible Years basic parent training; NBP, non-compliant behaviour problems; PTC, Parenting Tasks Checklist; NSDD, non-specific developmental disorders; P-ESDM, Parent Early Start Denver Model; ASD, autism spectrum disorder; MRB, managing repetitive behaviours programme; VIPP-AUTI, Video-Feedback Intervention to Promote Positive Parenting Adapted to Autism; PEQ, parental efficacy questionnaire; FBST, Functional Behaviour Skills Training; PPQ, Parent Perception Questionnaire; PTC, Parenting Tasks Checklist; PT, parent training; PCSSTP, Primary Care Stepping Stones Triple-P.

The majority of studies were conducted in Australia (*n* = 12), with three studies conducted in the UK, two studies each in Hong Kong, USA and New Zealand, while one study was conducted in each of the following countries: Portugal, Canada, Netherlands and Israel. The children’s ages ranged from 1 to 10 years. Eighteen studies had a mean children’s age younger than 5 years, while seven studies reported a mean age older than 5 years. Seven studies specifically recorded PSE scores of mothers; of these, six studies directed their interventions solely at mothers. The remaining 18 studies did not specify who received the intervention and they reported combined PSE scores, without stratifying the outcomes for mothers and fathers.

Parent training programmes were not standardised across studies. Of the better-known programmes, 15 studies assessed different forms of the Triple P-Positive Parenting Program©, two studies assessed the Incredible Years basic parent training programme, one tested the parent-administered version of the Early Start Denver Model and one tested Project ImPACT (Improving Parents as Communication Teachers). The remaining six studies trialled less commonly known interventions. Twenty-three studies had copyright or trademark licences for the interventions employed in the study. Furthermore, the interventions were administered either by psychologists (*n* = 12) or by healthcare or education practitioners (*n* = 13). These professionals included nurses, special education teachers and allied health professionals (such as speech and language therapists, occupational therapists and social workers).

The PSE levels were assessed using different measures: 17 studies used the PSOC, four studies used different formats of the PTC and the remaining four studies employed less commonly utilised PSE assessment tools.

## Treatment effects

### Summative parental self-efficacy measures (25 studies)

**FIGURE 3:**
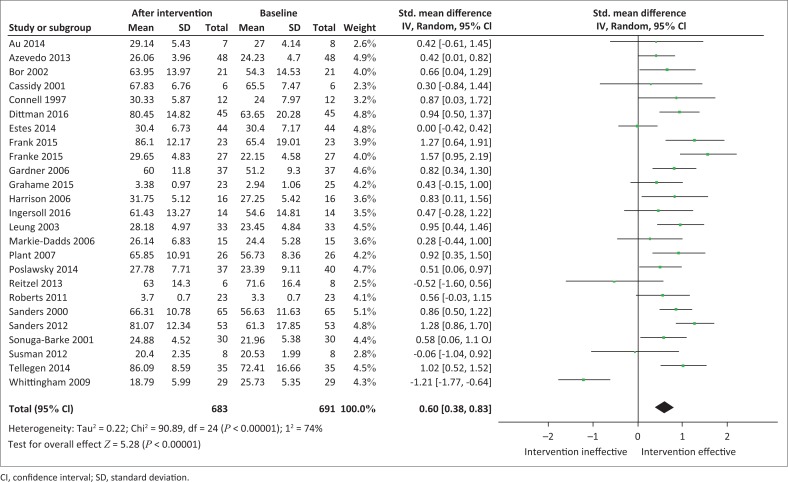
Random effects meta-analysis of the summative effects of parent training programmes on parental self-efficacy levels.

As displayed in Figure 3, compared to baseline measurements, parent training programmes resulted in a statistically significant increase in PSE levels across all studies, irrespective of assessment tool employed (*n* = 683; SMD, 0.60 [95% confidence interval {CI}, 0.38; 0.83]; *I*^2^ = 74%). Table 2 displays the summative results including those from the subgroup analyses.

**TABLE 2:** Summative parental self-efficacy outcomes and the subgroup analyses.

Subgroups analysed	*k*	Participants	*d* (Overall effect size)	*d* Lower 95% CI	*d* Upper 95% CI	*I*^2^
Summative PSE measures	25	683	0.60	0.38	0.83	74%
Children’s age						
5 years and older	7	160	0.34	−0.35	1.03	88%
Younger than 5 years	18	523	0.70	0.50	0.89	54%
Licencing						
Copyright	23	669	0.65	0.43	0.88	74%
Non-copyright	2	14	−0.26	−0.99	0.46	0%
Programme administrator						
Psychologist	12	385	0.53	0.16	0.90	84%
Health practitioner (other)	13	298	0.72	0.49	0.95	41%

*k*, number of studies; *d*, overall effect size; CI, confidence interval; *I*^2^, measure of degree of heterogeneity; PSE, parental self-efficacy.

### Subgroup analysis

#### Parental self-efficacy according to children’s ages

**FIGURE 4:**
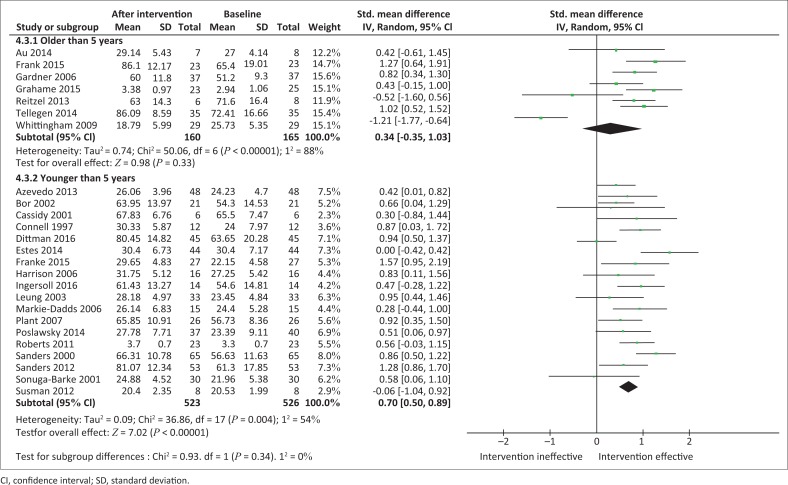
Random effects meta-analysis of the summative effects of parent training programmes according to child age.

Studies were stratified according to the mean ages of children in each study (Figure 4). Parents of children aged 5 years and older showed that the intervention had no statistically significant effect on PSE (*n* = 160; SMD, 0.34 [95% CI, –0.35; 1.03]; *I*^2^ = 88%). By contrast, parents of children younger than 5 years showed a statistically significant increase in PSE levels, thus favouring the intervention (*n* = 523; SMD, 0.70 [95% CI, 0.50; 0.89]; *I*^2^ = 54%).

#### Intervention type

**FIGURE 5:**
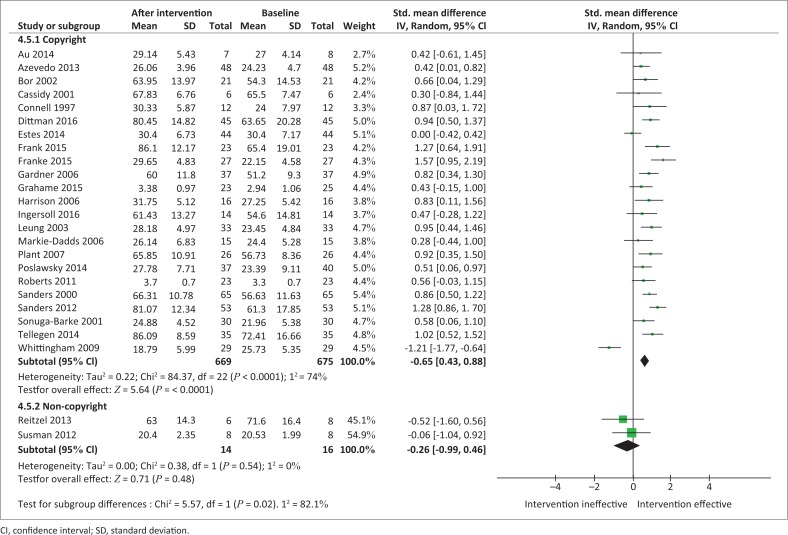
Random effects meta-analysis of the summative effects of parent training programmes according to programme type.

Studies were stratified according to whether they incorporated copyright or trademark interventions compared to non-licenced interventions (Figure 5). Copyright or trademark interventions showed a statistically significant effect for enhancing PSE levels (*n* = 669; SMD, 0.65 [95% CI, 0.43; 0.88]; *I*^2^ = 74%). In contrast, non-licenced interventions were ineffective for enhancing PSE levels and had an effect that was non-significant (*n* = 14; SMD, –0.26 [95% CI, –0.99; 0.46]; *I*^2^ = 0%).

#### Qualification of programme administrator

**FIGURE 6:**
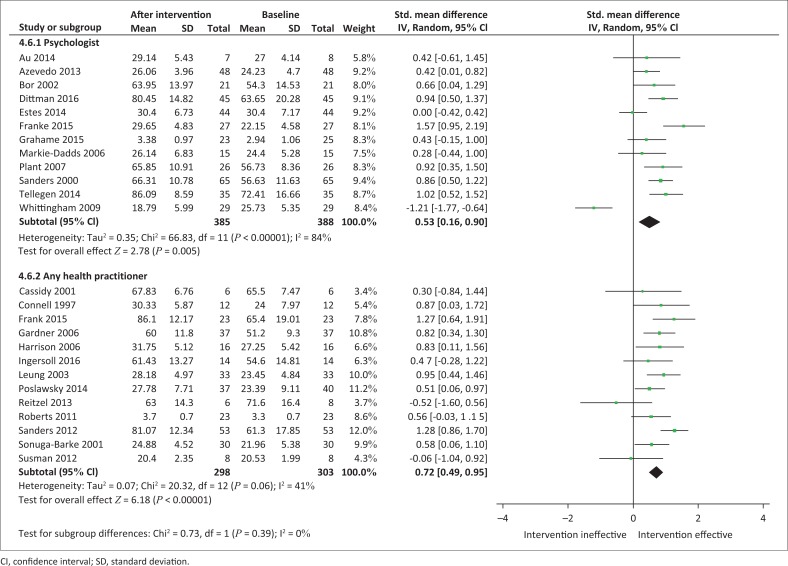
Random effects meta-analysis of the summative effects of parent training programmes according to professional delivering the intervention.

We considered whether studies implemented by healthcare practitioners other than psychologists showed variability in the effectiveness of the PSE outcomes compared to those that were facilitated by psychologists (Figure 6). Healthcare practitioners administering parent training programmes showed a statistically significant effect favouring the intervention (*n* = 298; SMD, 0.72 [95% CI, 0.49; 0.95]; *I*^2^ = 41%). Where psychologists administered parent training programmes, results also showed a statistically significant effect favouring the intervention (*n* = 385; SMD, 0.53 [95% CI, 0.16; 0.90]; *I*^2^ = 84%).

We used moderator analyses to assess the percentage of variability in the effect sizes across the parent training programmes for PSE in each subgroup analysis that was present. When exploring heterogeneity of the summative assessment for PSE measures, a substantial percentage of heterogeneity was present (*I*^2^ = 74%). Removing the study by Whittingham et al. (2009) reduced the heterogeneity to *I*^2^ = 52% and resulted in an increase in the effect size (*n* = 654; SMD, 0.70 [95% CI, 0.53; 0.87]; *I*² = 52%). In this study, 12 of the 29 children were diagnosed with Asperger’s syndrome, which may have resulted in children in this sample possessing relatively strong language abilities and milder difficulties with social interaction as compared to children with a diagnosis of ASD. Furthermore, 17 of the 29 parents did not seek help for their child’s emotional or behavioural problems, which suggests that these parents may have experienced relatively less stress than parents of children with ASD. Removing this study from the analysis meant that the remaining parents were a more homogenous group.

**FIGURE 7:**
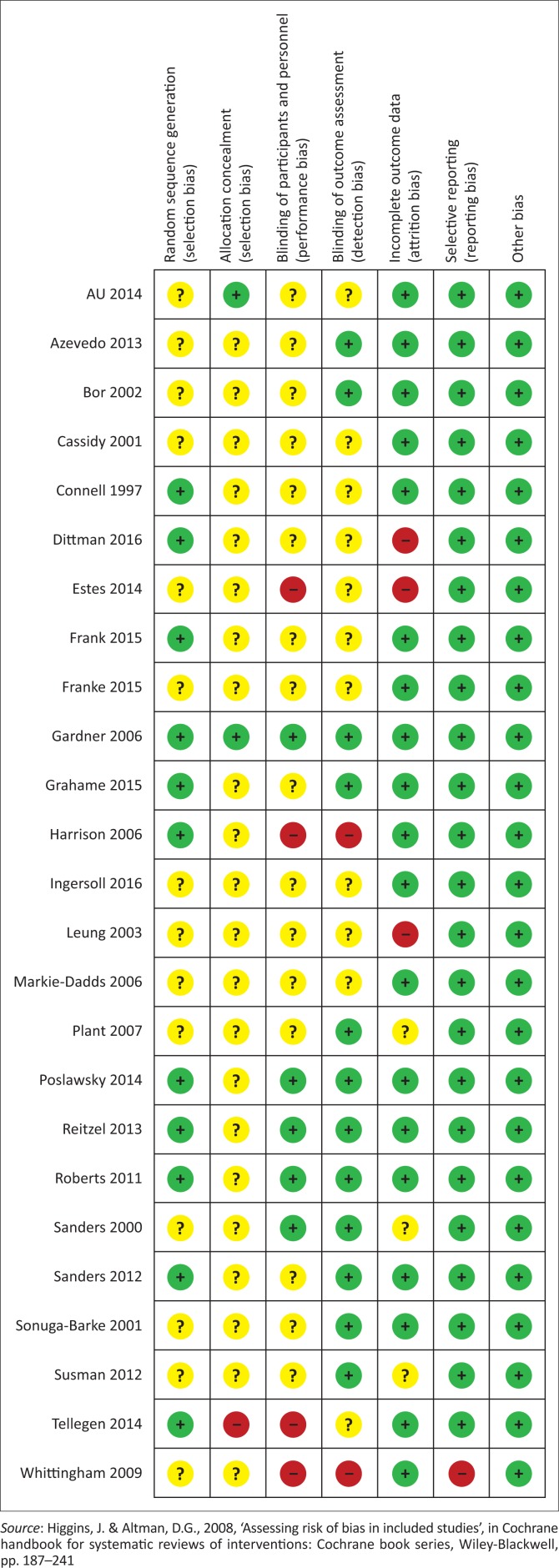
Risk of bias assessment for included studies according to Cochrane risk of bias tool.

A graphical representation of the risk of bias assessments is presented in Figure 7. Components assessing bias included blinding, allocation, incomplete outcome data, selective reporting and other potential sources of bias. The components were rated as being adequate, inadequate or unclear (Higgins 2008). The majority of the studies provided limited information regarding aspects of selection [specifically allocation concealment and sequence generation (randomisation)]. All of the included studies had a control group that consisted of no treatment or treatment as usual; therefore, blinding of participants to group allocation was not possible. Consequently, blinding of participants and personnel was the aspect that carried the highest risk of bias in the studies included in this review.

## Discussion

This systematic review found evidence for parent training programmes being effective in enhancing parental PSE levels. This finding was statistically significant and thus we are able to conclude that PSE is a robust parent outcome measure to evaluate the effectiveness of parenting programmes. Parental self-efficacy levels had a significant increase and large effect size (*d* = 0.60) for parents of children younger than 5 years of age, irrespective of the children’s diagnosis in the studies. Thus, data suggest that training parents of younger children are more beneficial in improving PSE outcomes than training initiated after the child is 5 years of age. The authors think that this may be because the skills taught to parents of younger children are based on developmental principles and consequently have a more direct impact on the developmental outcomes of children than skills taught to older parents. Parents who can see the positive impact that their newly acquired skill has on child outcomes would potentially be more likely to increase their belief (PSE) that they are able to provide the support that their child needs. These findings corroborate the increasing body of empirical evidence documenting the beneficial effects of early intervention on both parents’ and children’s outcomes (Guralnick 2017). These findings correspond to an earlier model that shows that heightened levels of PSE lead to subsequent heightened levels of success in the child (Ardelt & Eccles 2001). Thus, parenting programmes that increase PSE levels may also indirectly promote positive child outcomes (Ardelt & Eccles 2001; Coleman & Karraker 2003).

Parent training programmes were shown to be effective irrespective of whether they were administered by psychologists or other healthcare professionals. This finding may be of particular relevance in certain developing country contexts that do not have well-established professional training programmes for medical and allied health professionals and consequently may graduate a limited number of healthcare professionals on an annual basis. Task shifting has been suggested as a way to maximise access to interventions in contexts where there is a scarcity of trained professionals (Flisher et al. 2010; Rahman et al. 2008). In addition, there is an emerging body of evidence to suggest that alternative cadre professionals, such as rehabilitation care workers or community-based carers, are also able to effectively deliver parent training programmes (Flisher et al. 2010; Rahman et al. 2008; Reichow et al. 2013).

Finally, we wish to discuss the substantial amount of heterogeneity for the primary outcome measure. We employed the random-effects model throughout the analyses to account for this; however, in this meta-analysis, heterogeneity was particularly affected by one study. When removing the study by Whittingham et al. (2009), heterogeneity decreased considerably (the *I*^2^ value decreased from 74% to 52%) and the effect size increased. Heterogeneity in this study may also have been attributable to the high risk of performance and detection bias present in this study. Alternatively, we propose that the high number of children with Asperger’s syndrome (12 out of 29) included in this study compared to the other included studies may have affected the heterogeneity. Characteristics of children with Asperger’s syndrome include relatively strong language abilities and milder difficulties with social interaction relative to children with a diagnosis of autism.

We used the risk of bias tool as per PRISMA recommendation (Moher et al. 2009). Areas of bias that were underreported included performance bias, detection and attrition bias, including allocation concealment. Authors should pay attention to how they report participant selection and randomisation procedures, as well as how they report incomplete outcome data. These biases should be carefully considered in the design and implementation of future RCTs involving parent training programmes.

While there have been systematic reviews supporting the effectiveness of parent training programmes for parents of children with neurodevelopmental disorders, such as Skotarczak and Lee (2015) as well as Tellegen and Sanders (2013), this review is the first to evaluate the effect these interventions have in changing the PSE levels. No language limitations were set and articles not written in English were translated and included if they met the inclusion criteria. Furthermore, when investigating parent training programmes we chose to include all forms of parent training, rather than selecting specific programmes as other systematic reviews, such as Tellegen and Sanders (2013), have previously done. It is interesting to note that non-licensed interventions were ineffective in enhancing PSE levels. The authors postulate that licenced interventions have undergone a more rigorous development process than non-licensed interventions. This may result in stronger theoretical underpinning relating to both the development of the content and intervention activities, as well as a more detailed process of stakeholder engagement. This finding creates an interesting tension for researchers in LAMI settings interested in designing parent training programmes. Licensed interventions may be better at enhancing PSE, but they are not always contextually relevant and may need to be adapted to be socially acceptable in developing country contexts.

### Limitations of the study

One limitation of this review was the challenge we experienced in our efforts to provide summative estimates of the effectiveness of parent-based interventions, because of the varied nature (and poor description) of the different parenting interventions. In addition, numerous sources of bias were identified such as the fact that intention-to-treat analysis was not regularly used, which resulted in high levels of heterogeneity. We also acknowledge that these results only include PSE changes directly after intervention and do not include follow-up measurements of PSE. Furthermore, we acknowledge that our decision to include ADHD in this analysis of children with neurodevelopmental disabilities may receive criticism. However, recent research continues to highlight that ADHD and ASD share over 50% of their genetic factors (Van Steijn et al. 2012) and that two-thirds of individuals with ADHD display features of ASD (Mulligan et al. 2009). In this review, we collected PSE data that was measured subjectively using self-administered questionnaires. Nevertheless, self-report is typically the way in which this construct is measured in the field (Wittkowski et al. 2017). Lastly, it is still evident that none of the included studies was conducted in a LAMI country. As researchers in a developing country context, we view this as a significant constraint given the number of families in LAMI countries who have a child with a neurodevelopmental disability. Einfeld et al. (2012) conducted a review of interventions provided by parents. However, the authors feel that a systematic review of all of the caregiver skills-based interventions available in LAMI country contexts (irrespective of study design) would be helpful to obtain a clearer understanding of the existing evidence base and future research directions.

### Implications for practice

The results of the current systematic review present evidence that parent training programmes have a significant effect on the enhancement of self-efficacy levels for parents of children with neurodevelopmental disabilities. The data offers three insights for healthcare providers who provide parent training. This review suggests that parents of children younger than 5 years of age are most likely to report a change in PSE levels following parent training. Secondly, data from this review confirm licenced interventions to have greater benefits to PSE than non-licenced interventions. This is not surprising as interventions with copyrights or trademark licencing have traditionally been developed and refined over several years, and their development is usually supported by published evidence of their efficacy. The final clinical implication is that healthcare practitioners other than psychologists are successfully able to implement training programmes that enhance PSE. For those researchers who are interested in service delivery in developing country contexts, this finding is particularly important, given the dearth of suitably trained healthcare practitioners in LAMI settings able to provide children diagnosed with neurodevelopmental disorders, and their families, with appropriate care.

## Conclusion

As researchers within an African context, we recognise the need to pilot the efficacy of parenting interventions to change PSE levels in a LAMI context since, by the middle of this century, 40% of the world’s population of children will live in Africa (You et al. 2014). It is well known that Africa, as a continent, has limited access to resources and services to promote the health and development of its children. Therefore, it is important to consider how to reach the families of children with neurodevelopmental disabilities in these resource-constrained contexts. Consequently, we suggest that future research builds on this evidence base, which indicates that parents can be effectively trained by psychologists and allied health practitioners, by examining the effects of parent training provided by alternative cadre professionals.
